# Assessing Physical Health Risk in People With Intellectual Disability Using the Decision Support Tool for Physical Health [DST-PH]

**DOI:** 10.1192/bjo.2024.504

**Published:** 2024-08-01

**Authors:** Zoe Melrose, Catherine Rothon, Daanish Siddiqi, Ayodele Peters

**Affiliations:** South West London and St George's Mental Health NHS Trust, London, United Kingdom

## Abstract

**Aims:**

Accurately and comprehensively assessing physical health risk for people with intellectual disability (ID) is paramount in improving health outcomes, reducing the need for acute hospital admissions and preventing mortality. We aimed to compare the existing approach to assessing physical health risk with the use of a novel standardised risk stratification tool, the Decision Support Tool for Physical Health [DST-PH]. We hypothesise that DST-PH will be useful in improving and streamlining the assessment of physical health risk factors in people with ID.

People with ID are more likely to have poorer physical health outcomes and are at increased risk of premature and preventable death. Annual data from LeDeR (Learning from lives and deaths – People with a learning disability and autistic people) consistently underlines the need for developing strategies that reduce the risk of people with ID developing conditions associated with high causes of morbidity and mortality.

The DST-PH is an online tool that helps clinicians to identify people with ID who are at increased risk of early and preventable death. The tool captures key patient data about underlying health issues and risk factors that can contribute to poor health outcomes. Patients are then stratified according to their overall level of risk using a ‘RAG’ (red, green, amber) system. This allows targeted intervention and monitoring for those patients in need.

**Methods:**

All patient-facing staff in the Wandsworth Learning Disability Service were surveyed about their confidence levels in assessing physical health risk factors independently. We then asked each member of staff to assess physical health risk and assign a RAG rating for 2 randomly selected patients using their usual methods (clinical judgement). We then assessed the same patients using the DST-PH tool. Results were then compared to determine the degree of correlation between clinicians' existing risk assessment methods and the risk ratings assigned using the DST-PH.

**Results:**

Survey results showed that staff would welcome the introduction of a risk stratification tool. Comparison of risk assessment data showed a significant correlation between clinicians’ assessment and the results from the tool.

**Conclusion:**

Results evidenced the drive for ID clinicians to be observant of the physical health care needs of their patients. Introduction of the DST-PH may help to streamline the risk assessment process and increase confidence levels of clinicians.

